# Epidemiology of distal radius fractures: a detailed survey on a large sample of patients in a suburban area

**DOI:** 10.1186/s10195-022-00663-6

**Published:** 2022-08-30

**Authors:** V. Candela, P. Di Lucia, C. Carnevali, A. Milanese, A. Spagnoli, C. Villani, Stefano Gumina

**Affiliations:** 1 Dept. of Anatomical, Histological, Forensic Medicine and Orthopaedics Sciences, Sapienza University of Rome, Istituto Clinico Ortopedico Traumatologico (ICOT), Latina, Italy; 2 Dept. of Public Health and Infectious Diseases, Sapienza University of Rome, Rome, Italy; 3 Dept. of Anatomical, Histological, Forensic Medicine and Orthopaedics Sciences, Sapienza University of Rome, Umberto I Hospital, Rome, Italy

**Keywords:** Isolated distal radius fractures, Distal radius fractures epidemiology, Distal forearm fractures, AO classification, Distal radius fractures trauma mechanisms

## Abstract

**Background:**

Literature lacks data on correlations between epidemiology and clinical data of patients with distal radius fractures (DRFs).

**Aim:**

The aim of this study was to present a detailed epidemiologic survey of a large consecutive series of patient with DRFs.

**Materials and Methods:**

This retrospective study included 827 consecutive patients (579 females, 248 men) who sustained a DRFs in the last 5 years. All fractures were radiographically evaluated. DRFs were classified according to Association of Osteosynthesis classification. Data on age, gender, side, period in which fracture occurred, and fracture mechanism were collected. Statistical analysis was performed.

**Results:**

The patients’ mean age was 60.23 [standard deviation (SD) 16.65] years, with the left side being most frequently involved (56.1%). The mean age of females at the time of fracture was significantly higher than that of males.

The most frequent pattern of fracture was the complete articular fracture (64.3%), while the most represented fracture type was 2R3A2.2 (21.5%). Regarding the period in which the fracture occurred, 305 DRFs (37.5%) were observed in the warmer months and 272 (33.4%) in the colder months. Low-energy trauma occurring outside home was found to be the major cause of DRF throughout the year.

In both genders, trauma mechanism 2 was more frequent (59.4% F; 31.9% M; *p* < 0.01).

A bimodal distribution of fracture mechanisms was found in males when considering the patient’s age with a high-energy mechanism of fracture (3 and 4), identified in 21% (*n *= 52) of males aged 18–45 years, and a low-energy mechanism (1 and 2) was observed in 39.9% (*n *= 99) of males aged > 45 years. A significant correlation between all trauma mechanisms (from 1 to 6) and different fracture patterns (complete, partial, and extraarticular) was found (*p* value < 0.001). The mean age of patients with extraarticular fractures (mean age 61.75 years; SD 18.18 years) was higher than that of those with complete (mean age 59.84 years; SD 15.67 years) and partial fractures (mean age 55.26 years; SD 18.31 years). Furthermore, considering different fracture patterns and patient age groups, a statistically significant difference was found (*p* < 0.001).

**Conclusions:**

DRFs have a higher prevalence in females, an increase in incidence with older age, and no seasonal predisposition. Low-energy trauma occurring at home is the main cause of fracture among younger males sustaining fractures after sports trauma; Complete articular is the most frequent fracture pattern, while 2R3A2.2 is most frequent fracture type.

**Level of evidence:**

Level IV; case series; descriptive epidemiology study.

## Introduction

Distal radius fractures (DRFs) represent the most common fractures in adults, showing an overall prevalence of 17.5% with respect to all fractures [1]. Many factors have been proposed to determine the source of the increasing rates of DRFs: lifestyle [2], environment [3], rise in life expectancy [4], increased obesity in childhood [5], and osteoporosis rate [6] in elderly population. Previous research [8–11] has demonstrated that DRFs occur mainly in pediatric males and in postmenopausal women, while a consistent incidence has been observed also in young adult men aged 19–49 years [7]. High-energy trauma is the documented fracture mechanism in younger patients [8], while low-energy trauma, is the most common cause of injury in the elderly [2, 9, 10].

Little clarity emerges regarding the epidemiology of the fracture pattern [11] as DRFs are often identified with different eponyms, including Colles [12, 13], Smith [14], Barton [15], and Hutchinson fracture [16], instead of using a standardized classification system [7, 17], leading to uncertain clinical and radiological outcomes after both nonoperative and surgical treatments [18–20].

Although DRF is the most common fracture in adults, literature is still lacking in clinical data on several aspects of these fractures, such as the correlation between patient demographics, the fracture patterns, the period of the year in which the fractures occurred, and the different trauma mechanisms responsible for this injury.

The aim of this study was to present a detailed epidemiologic survey of a large consecutive series of patients with DRF in a large suburban area evaluating many aspects that are still unclear.

## Materials and methods

A retrospective study was conducted in the emergency department (ED) of a level I trauma hospital, serving a large suburban area. All the patients managed in the ED for a DRF in a 4-year period (from 1 January 2017 to 31 January 2021) were enrolled.

Patients with DRFs were identified from the clinical record, using the international statistical classification of diseases and related health problems, tenth version codes (ICD-10). A retrospective review of the clinical and radiological data of all patients was performed independently by three of the authors in order to collect information about age, gender, fracture side, and day of the week and period of the year in which fractures occurred. According to previous epidemiological studies [21, 22], six different trauma mechanisms were considered:

(1) low-energy trauma occurred in a public place; (2) low-energy trauma occurred at home; (3) sports trauma; (4) high-energy trauma resulting from car and pedestrian accident; (5) work-related injuries; (6) trauma resulting from assault, beatings, or theft.

Patients < 16 years old were excluded. We divided patients into three subcategories according to age: patients aged between 16 and 45 years; patients aged between 46 and 75 years; patients older than 76 years.

X-ray imaging, according to standard wrist trauma series consisting of a posteroanterior view, oblique view, and lateral view, was reviewed and DRFs were classified according to the Association of Osteosynthesis (AO)/OTA 2018 classification system [23] independently by three of the authors. If there was any disagreement, a consensus meeting with the senior author (S.G.) was undertaken.

## Statistical analysis

Continuous variables were expressed as median and interquartile range (IQR) or mean and standard deviation (SD) according to their distribution. Categorical data were recorded as frequencies and percentages. Comparisons between groups were performed by chi-square test or one-way analysis of variance (ANOVA). A post-hoc analysis with Bonferroni correction for multiple comparisons was performed.

## Results

During the study period, 1436 patients with DRFs (1005 F, 431 M; mean age 40.62 years) were admitted to the ED. There were 609 (42.4%) and 827 (57.6%) pediatric and adult patients, respectively. The study group was composed of 827 patients (579 F, 248 M; mean age 60.23 years; SD 16.65 years) since the pediatric population was not considered in the present study.

In the same period, 11,961 fractures in 12,054 adult patients were diagnosed. Maxillofacial or head fractures were not considered in the present study. DRFs represented 6.9% of the overall fractures in adult population.

Several associations with other fractures were found: in 12 cases DRF was bilateral (1.4%), and in 130 (15.9%) cases DRF was associated with ulnar styloid process fracture, in 67 (8.1%) cases with distal ulnar fracture, in 35 (4.2%) cases with scaphoid fracture, in 11 (1.3%) patients with ulnar diaphysis, in 13 (1.5%) cases with olecranon fracture, in 11 (1.3%) cases with proximal humerus fracture, and in 24 (2.9%) cases with femoral neck fracture. In 75 (9.1%) cases, an association with costal fracture was detected.

The mean age of females at the time of fracture was significantly higher than that of males [mean age 65.42 years (SD 13.18 years) in females and 48.11 years (SD 17.62 years) in males; *p* < 0.01]. Left side was most frequently involved (56.1%).

**Fig. 1:**
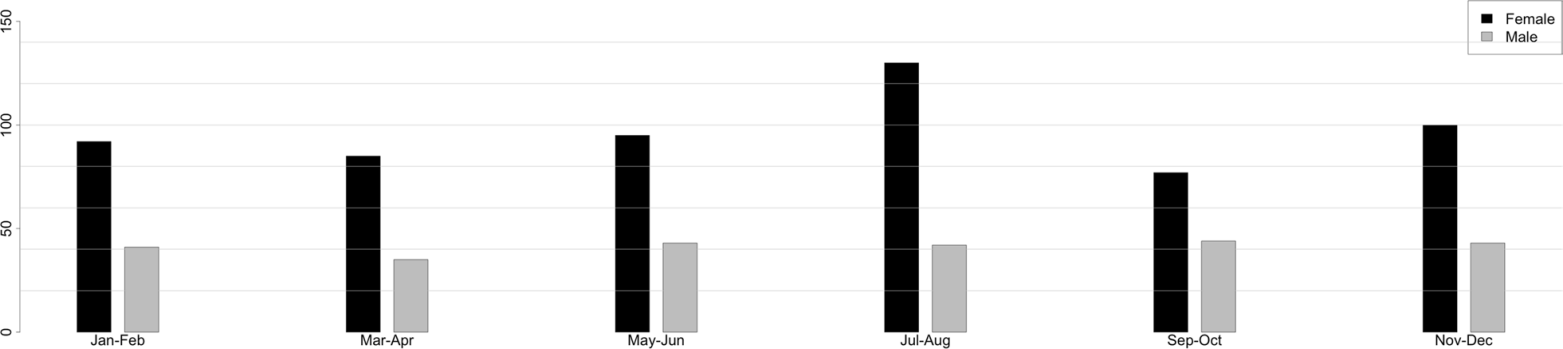
Yearly distribution of DRFs in both genders according to a 2-month classification period. DRFs, distal radius fractures

Figure 1 shows the yearly distribution of DRFs in both genders according to a 2-month classification period. Regarding the period of the year, 305 DRFs (37.5%) were observed in the warmer months (May to August) and 272 (33.4%) in the colder months (November to February). No significant differences were found (*p* = 0.85).

**Fig. 2:**
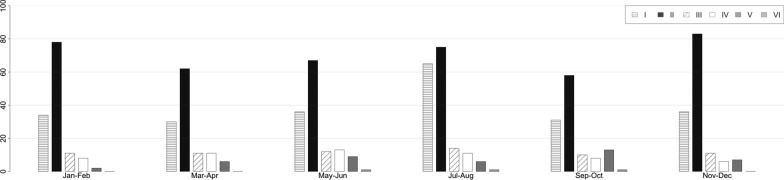
Yearly distribution of DRFs according to trauma mechanism in a 2-month classification period. DRFs, distal radius fractures. I, low-energy trauma that occurred in a public place; II, low-energy trauma that occurred at home; III, sports trauma; IV, high-energy trauma resulting from car and pedestrian accident; V, work-related injuries; VI, trauma resulting from assault, beatings, or theft

**Fig. 3:**
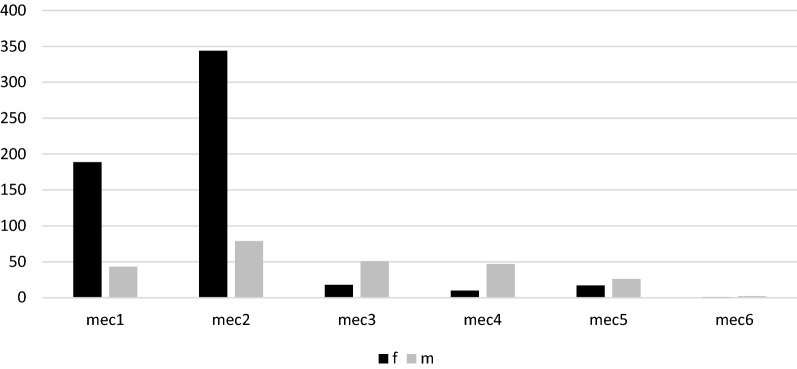
Distribution of DRFs in both genders according to the different trauma mechanisms. M, male; F, female. DRFs, distal radius fractures; Mec 1, low-energy trauma that occurred in a public place; Mec 2, low-energy trauma that occurred at home; Mec 3, sports trauma; Mec 4, high-energy trauma resulting from car and pedestrian accident; Mec 5, work-related injuries; Mec 6, trauma resulting from assault, beatings, or theft

Figure 2 shows the distribution of different trauma mechanism considering the different periods of the year; low-energy trauma occurring outside home was found to be the major cause of DRF throughout the year. Figure 3 shows the distribution of different trauma mechanisms according to gender. A statistically significant difference was found between gender in all trauma mechanisms except for 6. (*p* < 0.01). In both genders, trauma mechanism 2 was more frequent (59.4% F; 31.9% M; *p* < 0.01). The second most frequent mechanism in females was 1 (low-energy trauma at home; 32.6%), while in males it was 3 (sports trauma; 20.6%).

A bimodal distribution of fracture mechanisms was found in males when considering the patient’s age. A high-energy mechanism of fracture (3 and 4) was found in 21% (*n* = 52) of males aged 18–45 years, and a low-energy mechanism (1 and 2) was observed in 39.9% (*n* = 99) of males aged > 45 years. In females, mechanism 2 prevails in every age group.

**Fig. 4:**
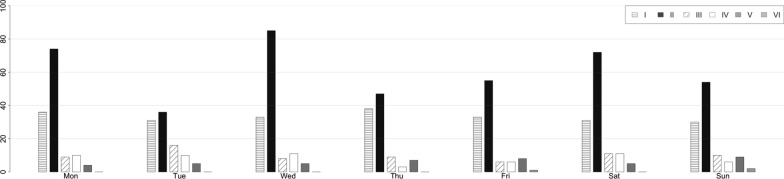
Distribution of trauma mechanisms according to the days of the week. I, low-energy trauma that occurred in a public place; II, low-energy trauma that occurred at home; III, sports trauma; IV, high-energy trauma resulting from car and pedestrian accident; V, work-related injuries; VI, trauma resulting from assault, beatings, or theft

Figure 4 shows the distribution of different trauma mechanisms according to the different days of the week. Low-energy trauma occurring outside home was found to be the major cause of DRFs during every day. No significant differences between gender were observed when the day in which DRFs occurred was considered (*p* = 0.027).

According to the AO/OTA classification system, Table 1 presents the different fractures patterns. The mean *κ* value for the intraobserver reliability assessment was 0.89 (95% CI 0.81–0.99), and according to the Landis and Koch criteria, it was considered as almost perfect agreement. The mean *κ* value for interobserver reliability was 0.67 (95% CI 0.59–0.75), and it was considered as substantial agreement according to the Landis and Koch criteria [21, 24].

**Table 1:** Fracture pattern according to AO/OTA distal radius fracture classification system

	**PATTERN AO**	***n*** **(%)**
	A1.1	2 (0.2)
	A2.1	39 (4.7)
Extra	A2.2	180 (21.5)
Articular	A2.3	23 (2.7)
	A3.1	1 (0.1)
	A3.2	2 (0.2)
	A3.3	10 (1.2)
	B1.1	17 (2.0)
	B1.2	1 (0.1)
Partial	B1.3	8 (1.0)
	B2.1	4 (0.5)
	B3.3	8 (1.0)
	C1.1	26 (3.1)
	C1.2	26 (3.1)
	C1.3	37 (4.5)
Complete	C2.1	171 (20.5)
	C2.2	35 (4.2)
	C2.3	45 (5.3)
	C3.1	89 (10.8)
	C3.2	95 (11.5)
	C3.3	8 (1.0)

**Fig. 5:**
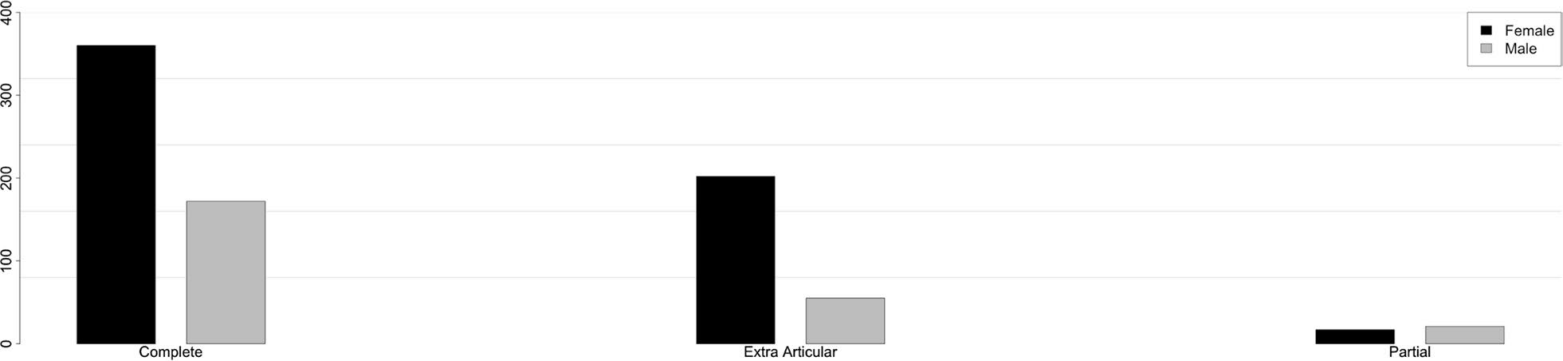
Distribution of DRFs patterns according to gender. M, male; F, female; DRFs, distal radius fractures

**Fig. 6:**
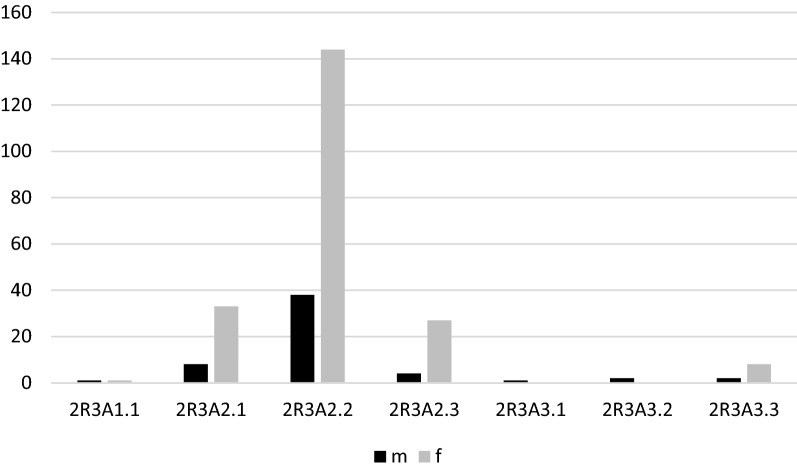
Distribution of extraarticular patterns of DRFs according to gender. M, male; F, female; DRFs, distal radius fractures

**Fig. 7:**
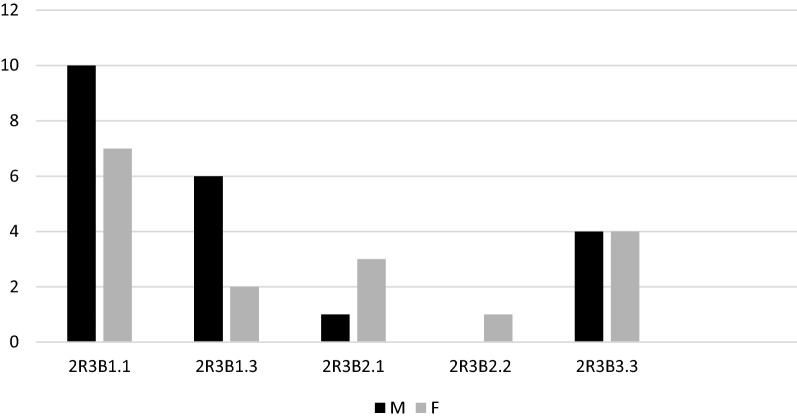
Distribution of partial-articular patterns of DRFs according to gender. M, male; F, female. DRFs, distal radius fractures

**Fig. 8:**
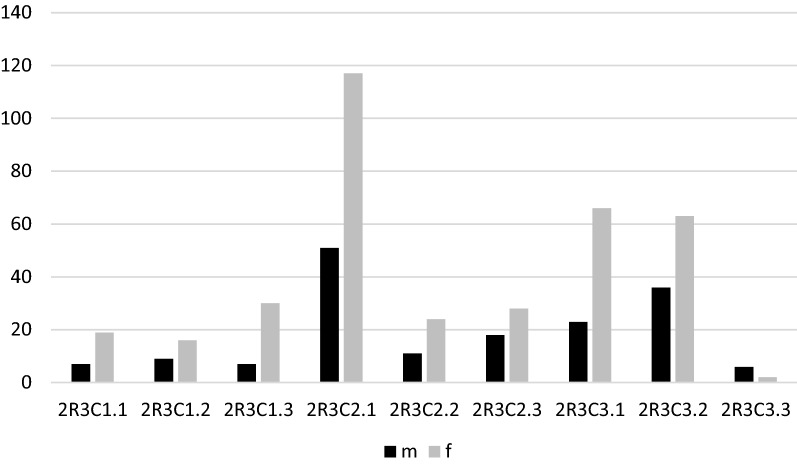
Distribution of articular patterns of DRFs according to gender. M, male; F, female; DRFs, distal radius fractures

The most frequent fracture group according to AO/OTA was the complete articular fracture (64.3%); however, the most frequent DRF type was 2R3A2.2. Figures 5, 6, 7 and 8 show the different distribution of DRF patterns according to gender. 2R3A2.2 was the most frequent fracture type in females (24.9%), while it was 2R3C2.1 in males (20.6%).

A post-hoc analysis of the standardized residues of the chi-square test with Bonferroni correction demonstrated a significant difference between genders in terms of extraarticular (*p* < 0.001) and partial fractures (*p* < 0.001), but no difference was found for complete ones.

A significant correlation between all trauma mechanisms (from 1 to 6) and different fracture patterns (complete, partial, and extraarticular) was found (*p* < 0.001).

Considering the association between different pattern of DRFs and the different periods of the year (two-classification month), no significant differences were found. No significant association between fracture type and days of the week was found (*p* = 0.76).

The mean age of patients with extraarticular fractures (mean age 61.75 years; SD 18.18 years) was higher than those with complete (mean age 59.84 years; SD 15.67 years) and partial fractures (mean age 55.26 years; SD 18.31 years). Furthermore, considering different fracture patterns and patient age groups, a statistically significant difference was found (*p* < 0.001).

## Discussion

Several studies focused their attention of the epidemiology of DRFs [8, 10, 25]. However, some methodological weaknesses emerge; in 1999, Lindau et al. [8] performed an epidemiologic survey of 341 patients with DRF living in Sweden. Unfortunately, only young adults were considered, and all data were obtained from registries with no fracture classification. The same age limitation is present in a study by Diamantopoulos et al. [25], which considered only middle-aged and elderly population. We performed a detailed epidemiological survey on a large group of patients living in a suburban area. All patients aged > 16 years old were evaluated, and DRFs were classified according to AO/OTA by a single center, contrarily to previous research [8, 10].

In our series, the prevalence of DRFs was 6.8% with respect to all fractures in adult population, representing the most frequent fracture followed by femoral neck (6.2%) and proximal humerus (5.4%).

Owing to the unpredictability of trauma, no differences according to the side of DRFs were found in our series. As previously described [2, 26, 27], our results confirmed that DRFs were more frequent in females (ratio 3:1) and that the mean age of females was considerably higher; these results may be explained by the higher susceptibility to osteoporosis [21] and longer life expectancy in females.

No difference was found regarding the period of the year in which the fracture occurred, in contrast to previous studies [3, 9, 10, 25, 27] that documented a higher incidence of DRFs during colder months. All previous research was performed in Northern Europe, and the authors justify the higher incidence of DRFs during winter months with the climatic conditions and the fewer hours of sunlight that may increase the risk of accidents. Furthermore, previous epidemiological surveys were performed in areas that probably depopulate in the summer period. In our sample, no monthly differences were found, probably because in Southern Europe no severe climatic conditions are present in the winter months and also because the inhabitants of the area served by the ED remain constant throughout the year.

We found that the most frequent DRFs pattern was complete articular (64.3%) according to AO/OTA classification, with no difference between genders. As described by Clayton et al. [28], a relationship between poor bone quality and severity of DRFs was demonstrated. In our series, DRFs occurred mainly in elderly patients, who are more exposed to osteoporosis, and this maybe the reason for the higher prevalence of the most severe DRF pattern. In contrast, in our sample, the prevalence of extraarticular fractures was significantly higher in females, which may be due to males being more prone to sustaining DRFs following high-energy trauma.

We found that the most common traumatic mechanism was low-energy trauma considering the whole studied population, as previously reported [2, 9, 10]. However, considering gender, differences emerged: low-energy trauma mechanism, occurring at home, was found to be the second mechanism responsible for DRFs in females but not in males, who sustained DRFs due to trauma related to sports and street accidents. This finding might be related to the fact that males have a greater aptitude for contact sporting activities and cycling compared with females.

In our sample, a stable distribution of different trauma mechanism was observed, without any distinction between working days and weekend, except for DRFs resulting from street accidents, which occur more frequently during working days; this was predictable and may be explained by the higher amount of traffic.

The current study has some limitations. Fracture classification was based only on X-ray images. CT scans for DRFs was performed only in case of displaced comminuted fractures for preoperative planning in the cases in which X-ray imaging was not sufficient for the choice of treatment and not as a classification tool.

## Conclusion

This epidemiologic study, conducted on a large number of people with DRFs, confirmed a higher prevalence in females, an increase in incidence with older age, and that no seasonal predisposition exists. In addition, the study showed that (1) low-energy trauma occurring at home is the main cause of fracture, with younger males sustaining fractures after sports trauma; and (2) complete articular is the most frequent fracture pattern, while 2R3A2.2 is the most frequent fracture type.

## Data Availability

Submitted as supplementary material.
